# *Salmonella enterica* Infections in the United States and Assessment of Coefficients of Variation: A Novel Approach to Identify Epidemiologic Characteristics of Individual Serotypes, 1996–2011

**DOI:** 10.1371/journal.pone.0145416

**Published:** 2015-12-23

**Authors:** Amy L. Boore, R. Michael Hoekstra, Martha Iwamoto, Patricia I. Fields, Richard D. Bishop, David L. Swerdlow

**Affiliations:** 1 Epidemic Intelligence Service, Scientific Education and Professional Development Program Office, Centers for Disease Control and Prevention, Atlanta, Georgia, 30333, United States of America; 2 Division of Foodborne, Waterborne, and Environmental Diseases, Centers for Disease Control and Prevention, Atlanta, Georgia, 30333, United States of America; California Department of Public Health, UNITED STATES

## Abstract

**Background:**

Despite control efforts, salmonellosis continues to cause an estimated 1.2 million infections in the United States (US) annually. We describe the incidence of salmonellosis in the US and introduce a novel approach to examine the epidemiologic similarities and differences of individual serotypes.

**Methods:**

Cases of salmonellosis in humans reported to the laboratory-based National *Salmonella* Surveillance System during 1996–2011 from US states were included. Coefficients of variation were used to describe distribution of incidence rates of common *Salmonella* serotypes by geographic region, age group and sex of patient, and month of sample isolation.

**Results:**

During 1996–2011, more than 600,000 S*almonella* isolates from humans were reported, with an average annual incidence of 13.1 cases/100,000 persons. The annual reported rate of *Salmonella* infections did not decrease during the study period. The top five most commonly reported serotypes, Typhimurium, Enteritidis, Newport, Heidelberg, and Javiana, accounted for 62% of fully serotyped isolates. Coefficients of variation showed the most geographically concentrated serotypes were often clustered in Gulf Coast states and were also more frequently found to be increasing in incidence. Serotypes clustered in particular months, age groups, and sex were also identified and described.

**Conclusions:**

Although overall incidence rates of *Salmonella* did not change over time, trends and epidemiological factors differed remarkably by serotype. A better understanding of *Salmonella*, facilitated by this comprehensive description of overall trends and unique characteristics of individual serotypes, will assist in responding to this disease and in planning and implementing prevention activities.

## Introduction

Salmonellosis is a nationally notifiable disease in the United States (US), where it causes an estimated 1.2 million infections and 450 deaths annually [1]. *Salmonella enterica* subsp. *enterica* serovars, of which more than 2,500 have been identified with new additions continuously added, are adapted to a variety of hosts. Some serotypes have but one or a few hosts, whereas others have many [2]. Serotype Typhi, for example, is known to exist only in humans, serotype Choleraesuis has a primary reservoir in pigs, and serotype Dublin in cattle [3–6]. Serotype Typhimurium can be found in the gastrointestinal tracts of many animal species and has even been shown to replicate in the tissues of some plants [7]. Salmonellosis in humans can result from eating inadequately cooked animal-derived foodstuffs that harbor the bacteria, produce, other food items contaminated from animal sources, consumption of contaminated water, or by direct or indirect exposure to reptiles, amphibians, other infected animals, or humans [8–26]. Different serotypes tend to be associated with particular routes of infection based on their natural ecology. Serotype Enteritidis, for example, is widespread among egg-laying poultry and has caused multiple outbreaks of illness associated with shell eggs [9,27,28]. Serotype IV 48:g,z51:− causes sporadic illness from contact with the environment of a marine iguana, the serotype’s only known host [29]. The ecology of many serotypes is still unknown. A better understanding of the ecologic niches of common serotypes is needed; however, the number and diversity of serotypes makes gaining such understanding difficult.

We describe the trends in cases of *Salmonella* infection in humans reported to the Centers for Disease Control and Prevention (CDC) during 1996–2011 in the US. As a first step toward understanding changes over time and identifying and classifying unique ecologic niches of common serotypes, we introduce a way of ordering serotypes by variability of incidence rates across values of key epidemiological variables using simple coefficients of variation. The idea of this organization scheme is to allow for the identification of serotypes with highly skewed rate distributions, suggesting a particular reservoir or mode of transmission. A better understanding of the epidemiology of *Salmonella* serotypes and ecologic niches of the more common ones would help researchers target control efforts.

## Methods

### Ethics Statement

The National Center for Emerging and Zoonotic Infectious Diseases within the Centers for Disease Control and Prevention determined that this investigation did not meet the definition of research as provided by 45 CFR 4 6.102(d) and therefore Institutional Review Board review was not required. This decision was made as only existing public health surveillance data,that is completely anonymized with no possibility of being linked directly or indirectly to human subjects, were used.

### Data Source

National *Salmonella* surveillance in the US was established in 1962. Reports of *Salmonella* cases are made to CDC via a passive, laboratory-based surveillance system conducted in 50 states and the District of Columbia. Clinical laboratories are required or requested to send human *Salmonella* isolates to state and territorial public health department laboratories for serotyping. Isolates are serotyped according to the Kauffman and White scheme [2], and then the health departments forward serotype information, date of specimen collection, source of specimen (eg stool, blood, etc), and patient demographics (age, sex, race, state and county of residence) to CDC electronically. CDC summarizes and publishes these data annually. This publication is the only source of nationally-acquired information about *Salmonella* serotypes (details on the surveillance system have been previously published) [30,31]. We analyzed 1996–2011 data for all 50 states and the District of Columbia (DC) in describing overall and regional change in incidence over time. Data from five states (Montana, Texas, Florida, Nebraska, and Wyoming) and DC were excluded from further analyses because reports from these states had missing or partial serotype data for >10% of *Salmonella* isolates for eight or more of the 16 years of surveillance. Data on race and county of residence were not analyzed due to missing information and reporting inconsistencies among locations.

### Analysis

We calculated incidence rates for overall *Salmonella* infection (all reported cases) and for subgroups defined by reported serotype, date specimen was obtained (by year and by season), source of specimen, age, sex, and state of residence of patient. We also calculated incidence rates for these subgroups within groups defined by reported serotype. Blood, bone, joint, and cerebrospinal fluid were considered sterile sources, while stool, abscesses, sputum, and other sources were considered non-sterile sources. US census data were used to estimate US and state population sizes for calculating incidence rates. Rates were examined by month of the year and also grouped into “seasons” defined as December-February, March-May, June-August, and September-November. Ages were aggregated to align with US census age groups. For geographic analysis, states of residence were aggregated by census regions (Northeast, South, West, and Midwest– www2.census.gov). To look at changes in overall and serotype-specific incidence, we also aggregated year into early (1996–2003) and late periods (2004–2011); the even split of time used for simplicity and for a more robust comparison, especially for less-commonly reported serotypes.

We included all isolates reported from states in our analysis of general trends. Other factors were analyzed overall and by serotype. For serotype-specific analyses, only those 37 serotypes with at least 1,600 reported isolates for the 16 years were included—and these were considered common serotypes. This restriction was made to limit potential bias due to possible inaccuracies in the serotyping of less common serotypes at state laboratories. For example, proportions of unknown and partially serotyped isolates varied by state. Counts for serotypes I,4,[5],12:i:− and Typhimurium (including Typhimurium var. 5-) were combined (and labeled Typhimurium^+^) because not all state laboratories could make the distinction. Counts for serotypes Paratyphi B and Paratyphi B var. L(+) tartrate + were also combined (and labeled “Paratyphi B var. L(+) tartrate +”) because serotype Paratyphi B tartrate− is rare in the US [32].

We compared serotype incidence rates with respect to their dependence on the key variables of state of residence, month, age group, and sex. We did this by using coefficient of variation (CV) as the measure of rate dispersion. CV is defined as the ratio of the standard deviation (σ) to the mean (μ). CV is reported as a percentage [CV = (σ/ μ) * 100%] and does not depend on the size of the mean. It follows, therefore, that specific characteristics of serotypes, even higher and lower incidence serotypes such as Typhimurium^+^ and Norwich, can be directly compared. By definition, a CV of 0% indicates a constant rate across categories, such as state or month. Higher values (high standard deviations [σ] compared with the mean) indicate greater dispersion, and values exceeding 100% tend to indicate positive skewness of rates. For example, suppose 10 months have very similar rates, but the two remaining months have rates that are 5 times higher. Such rates would show positive skewness and a high CV.

We used incidence rates when calculating CV across state, age, and sex to control for population variation. The CV by month was calculated using isolate counts because, with person-time constant, the result is the same as using incidence. We ranked serotypes for various epidemiological factors by CV. We also examined the degree to which serotypes with high or low ranking on one factor corresponded to serotypes with high or low ranking on another. We used Spearman’s rank correlation to make these comparisons, testing the hypothesis that factor CV did not depend on serotype [33]. Actual rank correlations are not reported.

## Results

### Changes Over Time and Epidemiological Characteristics

**Fig 1:**
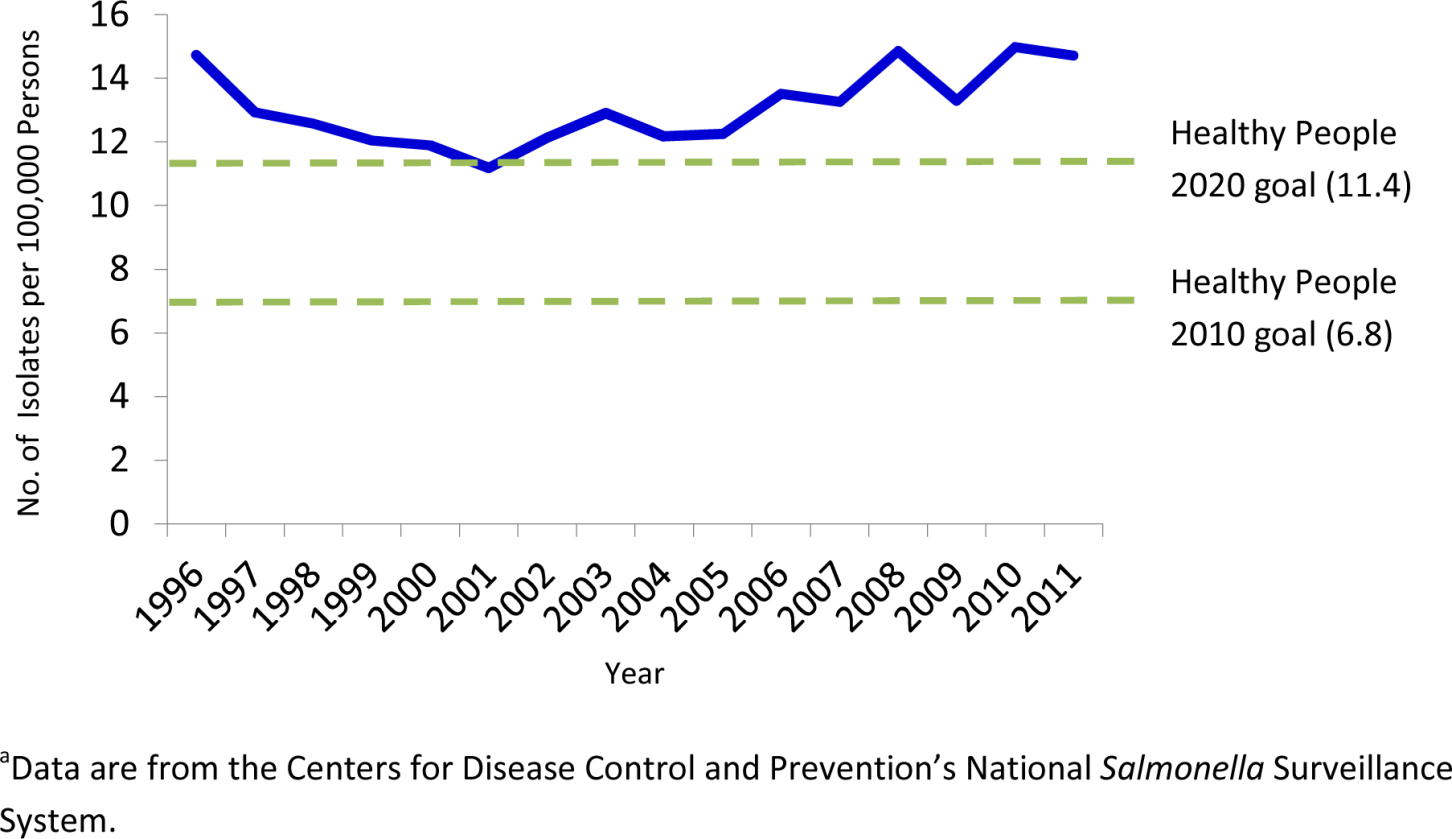
Incidence of *Salmonella* Isolates from Humans, United States, 1996–2011.

During 1996–2011, a total of 608,571 *Salmonella* isolates from humans were reported to CDC, which averages 38,000 isolates per year. The average annual rate of isolation was 13 cases/100,000 persons, with the highest rate (15) in 2010 and the lowest (11) in 2001 (Fig 1). The overall average for the latter half of the 16 year period (2004–2011 at 14 cases per 100,000) was 9% higher than in first half (1996–2003, 13 cases per 100,000). A total of 1,280 serotypes were reported, although 416 were reported only once. Serotype data were not reported for nearly 37,000 (6% of total) isolates, and 20,060 (3%) were only partially serotyped.

The Northeast US had the highest rate of reported *Salmonella* cases, with an incidence of 15 cases/100,000 population. The South had an incidence of 14, the West 13, and the Midwest 11 cases/100,000. The South was the only region in which the incidence increased; the average rate during 2004–2011 was 34% higher than during 1996–2003. The Northeast, West, and Midwest regions experienced a decrease in the latter period (4%, 6%, and 6% decreases, respectively). National incidence was highly seasonal, with the highest percentage of cases reported in July and August (13% each) and the lowest in February (5%).

**Fig 2:**
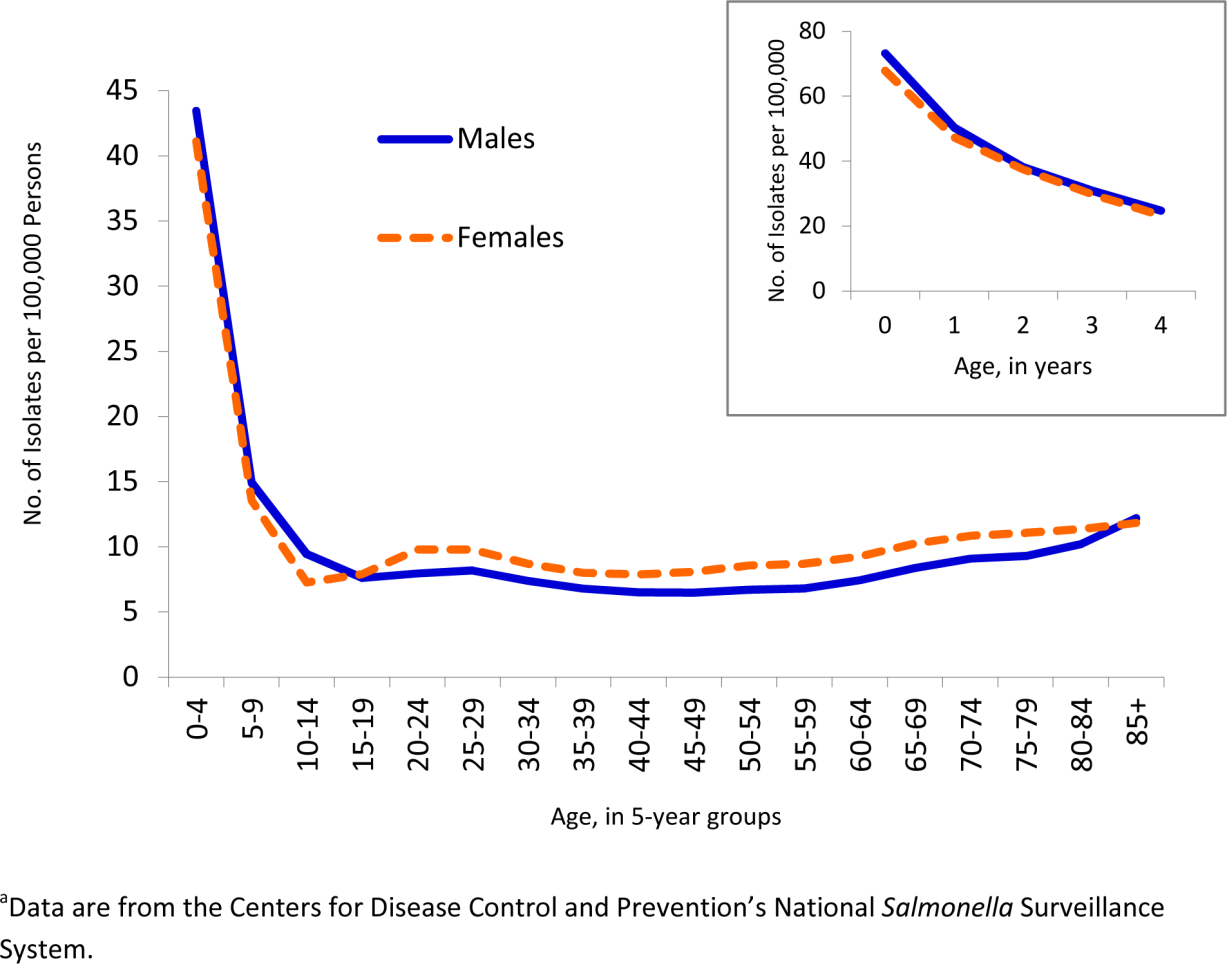
Incidence of *Salmonella* Isolates from Humans by Age Group and Sex, United States, 1996–2011, with Outset Graphs Highlighting 0–4 Year-Old Group.

The overall incidence of *Salmonella* infections was highest among young children (i.e., younger than 5 years), at 45 cases/100,000 population (27% of all reported isolates with known age). The 5- to 10-year-olds had the next highest rate, at 15, followed by the 85+ year-old group, at 13. The lowest rate was among 40- to 44-year-olds, at 8. Boys younger than 15 years had higher incidence than girls of the same age (23 vs 21) but otherwise males averaged a 10% lower incidence than females (Fig 2).

Few isolates (6%) were obtained from sterile sources (primarily blood, joints, spinal fluid). Young children had the highest rates of sterile-site isolation at 2/100,000. Among those over 4 years, rates of sterile-site isolation increased with age from 0.6/100,000 in children aged 5 to 9 years to 1/100,000 in adults aged 85 or older. Males had rates of sterile-site isolation 6%-82% higher than females in all age groups, and the disparity increased with age.

### Changes by Serotype

**Fig 3:**
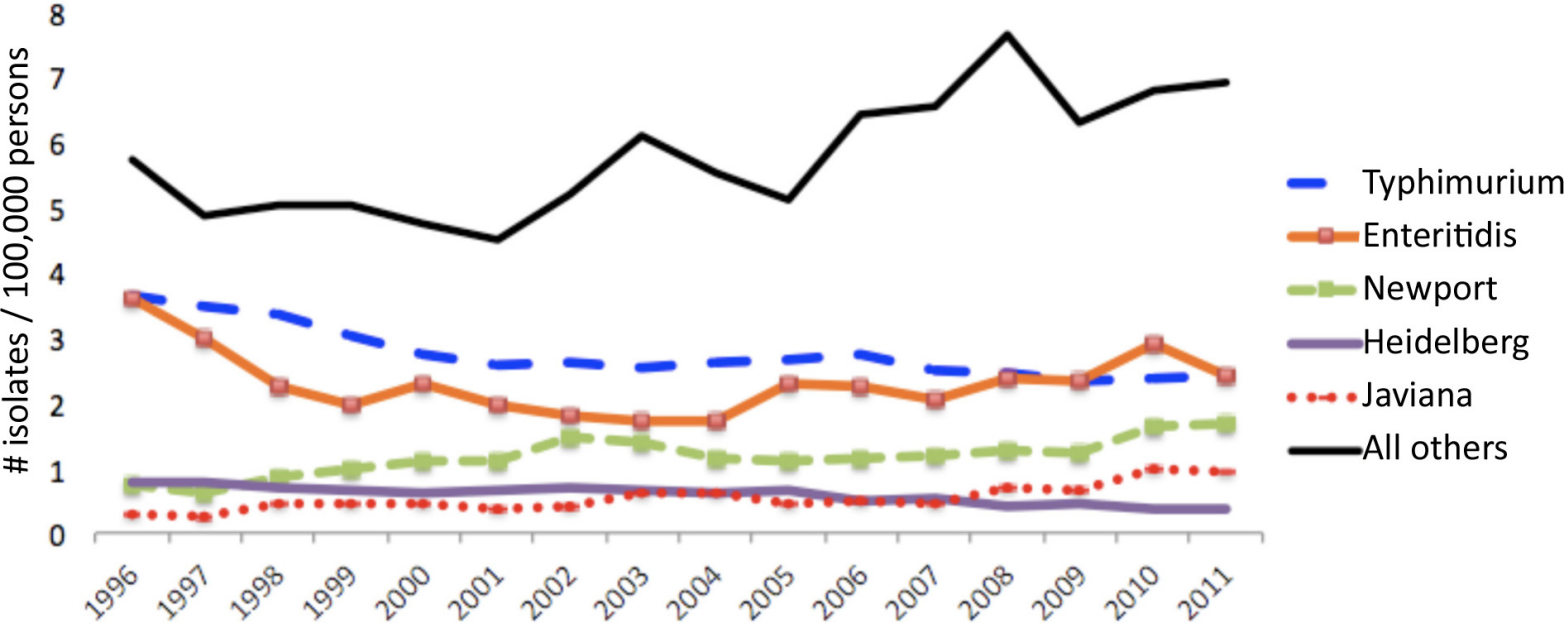
Incidence of five most common *Salmonella* serotypes reported to CDC, United States, 1996–2011.

Thirty-seven serotypes were each reported at least 1,600 times during 1996–2011. The five most commonly reported serotypes, Typhimurium^+^, Enteritidis, Newport, Heidelberg, and Javiana, accounted for 62% of fully serotyped isolates. The average incidence of infection for serotypes Typhimurium^+^, Enteritidis, and Heidelberg decreased in the second half of the study period compared with the first half (16%, 1%, and 30% decreases, respectively), whereas serotypes Newport increased 27% and Javiana increased 63% (Fig 3). Other serotypes with notable increases in incidence included Saintpaul (43% increase, largely due to an outbreak in 2008) and Mississippi (63% increase) (S1 Table).

### Relative Dispersion of Serotypes Across Key Variables

CVs for 37 serotypes examined by state, month, age group, and sex are shown in S1 Table. The serotypes with the most evenly distributed rates across all states and regions were Typhimurium^+^ (CV 30%), Infantis (CV 40%), and Heidelberg (CV 33%). At the other end of the spectrum, serotypes Mississippi (CV 256%), Rubislaw (CV 197%), and Give (CV 195%) were heavily concentrated in the Gulf Coast states. Other serotypes that showed geographic concentration included Norwich (CV 161%), being reported mostly from the lower Midwest into the South, and Javiana (CV 135%), also most frequently reported from the South.

Serotypes with the largest CVs by month were Norwich (CV 87%), Javiana (CV 83%), Mississippi (CV 69%), and Newport (CV 68%). All were highly concentrated in months that are generally warmer in the US, averaging 45% of reported isolates of these four serotypes (range, 41% to 47%) in summer (June-Aug) and 8% (range, 7% to 11%) in winter (Dec-Feb). Conversely, serotypes Senftenberg, Mbandaka, Anatum, and Derby had low CVs by month (18%, 21%, 24%, and 24% respectively), indicating that isolations occurred fairly evenly throughout the year, with an average of 30% (range, 28% to 31%) of isolates reported in summer and 22% (range, 20% to 26%) in winter.

The variation in incidence rate by age was highest in serotypes Rubislaw (CV 265%), Mississippi (CV 160%), Poona (CV 151%), and Schwarzengrund (CV 150%)—all found mostly in young children. Enteritidis (CV 24%), Berta (CV 40%), and Braenderup (CV 41%) were the serotypes most equally distributed by age. The rate was highest among young children for 34 of the 37 common serotypes; the exceptions were Senftenberg (highest rates among adults aged 70 years and older), Paratyphi A (highest rates among 20- to 34-year-olds), and Tennessee (highest rate among adults aged ≥74 years).

Most serotypes were either equally distributed between men and women or showed a slightly increased rate in women. Serotypes with the greatest incidence variation were Tennessee (CV of 54%, 69% of isolates from women), Senftenberg (CV 35%, 62% of isolates from women), and Anatum (CV 28%, 60% of isolates from women). The most evenly distributed serotypes, with nearly 50% of isolates from each sex, were Heidelberg (CV 0.3%), Sandiego (CV 0.5%), and Schwarzengrund (CV 0.6%).

Serotypes with higher variation in incidence by age group (ie, skewed toward particular ages) were more common among males (Spearman’s correlation *p* <0.01) and had higher incidence variation by state (*p* <0.01). There was no correlation between variation by age and season (*p* = 0.13), sex and season (*p* = 0.74), or sex and region (*p* = 0.36). Serotypes whose incidence increased the most in the second half of the 16-year study period had higher incidence variation by season (Spearman’s correlation *p* <0.01) and by state (*p* = .02). Changes in incidence did not correlate with variation by age or sex.

## Discussion

This study introduces a simple way to describe the diversity of common *Salmonella* serotypes. Control of *Salmonella* infections is difficult because of the number and diversity of *Salmonella* serotypes. Each may have unique reservoirs, modes of transmission, and epidemiology. Trends in incidence over time differed remarkably by serotype, as did incidence by age group, seasonality, and geography. National surveillance data show the overall incidence rates of all reported *Salmonella* infections in the US did not change significantly during the study period and remain well above Healthy People goals to reduce infections [34]. While the incidence of the most commonly reported serotype, serotype Typhimurium, decreased over time, this positive trend was countered by the increases in other serotypes such as Newport and Javiana. Control and prevention measures are needed that target particular serotypes.

Our finding that four of the five most common serotypes had low CVs with respect to state indicates that these serotypes have a relatively even geographic distribution. This may suggest homogeneous sources of infection that are widely dispersed, such as nationally distributed food products, or an even mix of multiple sources of infection. In contrast, Javiana, the fifth most commonly reported serotype, was among the most geographically restricted and found predominantly in the Southeast. A serotype is likely to be restricted geographically if tied to a natural reservoir with a restricted range or to local habits or food products. Javiana’s association with amphibians might explain part of its geographic distribution [22]. Other serotypes restricted to the South include Mississippi, Norwich, and Rubislaw, which also had among the greatest increases in incidence during the 16 years (63%, 50%, and 32%, respectively). This finding is consistent for the South census region, the only region to report an overall increase in *Salmonella* incidence. Maurer *et al* recently reported several serotypes known to be associated with human illness can be found widely distributed in wildlife and water sources in the Southeastern state of Georgia [35]. Because of the increased incidence in the South and in serotypes found mostly in the South, more attention is needed to identify such reservoirs and routes of transmission unique to the area so control measures can be tailored.

The serotypes with the highest variation across months were concentrated in the southeast (serotypes Javiana, Mississippi, and Bareilly). A strong concentration in a particular time of year could indicate an increased presence of a host (eg, amphibians, reptiles), increased human exposure to the source, or increased contamination in an otherwise omnipresent source.

Understanding the distribution of serotypes across age and gender is a step toward better understanding the natural ecology of serotypes. Serotypes Enteritidis, Berta, and Braenderup had the lowest variation by age group, which may reflect unvaried sources of infection or an even mix of sources. Serotypes Rubislaw and Mississippi had highest variation by age group and were seen almost exclusively in young children. They were also among the few serotypes with a slightly higher rate in males (53% each). Reller *et al* noted that serotypes with male predominance tended to be isolated from children (younger than 19 years), whereas those with female predominance were more often isolated from adults (older than 20 years) [36]. These patterns may offer insight into the natural reservoirs and exposure risks. For example, reptile-associated serotypes have been shown to disproportionately affect young boys [21,29]. The finding that serotypes restricted by age were also restricted geographically and more common in boys may reflect a natural reservoir in reptiles or amphibians. *Serotypes* more common in older age groups were also more common in women. Further, salmonellosis is observed more frequently among women than men in the mid- to older-age groups, a trend speculated to be partly due to, among other factors, food preparation exposures and exposure to fresh produce [36]. Infections from contaminated fresh produce are increasingly common in the US [13]. In a population survey, women reported eating more produce than men [37], and investigations of produce-related outbreaks frequently find more women affected than men [14,16,17,38,39]. Host susceptibility and unrecognized serotype-specific factors (eg, those affecting the urinary tract), may partially explain these trends.

This study has several limitations. Availability of resources for serotyping and reporting cases of *Salmonella* vary by state and county and change over time. Trends and geographic clustering of serotypes could be affected by this differential reporting. The overall increase in the percent of isolates for which no serotype or only partial serotype data were reported, from 5% in 1996 to 12% in 2011, impairs our ability to accurately track trends by serotype. The exclusion of five states and DC due to a high percentage of missing or incomplete serotypes similarly impairs serotype-specific analysis and could introduce bias depending on the specific trends or demographics in those states. Missing values for age, sex, and source could introduce bias if those missing values were also more likely to occur for certain serotypes or other paramaters examined in the study. In addition, for every reported case of *Salmonella*, an estimated 28 cases go unreported. This means that our understanding of *Salmonella* is based on observations from a small, select portion of cases [1]. For example, less underreporting occurs among young children compared with other age groups, so trends are weighted toward the experience of young children or people with severe illnesses who are more likely to seek care [40]. However, general and serotype-specific trends over time would not be differentially affected, and an active surveillance system for *Salmonella* (FoodNet) found that the data obtained by the passive nationwide surveillance system was relatively complete and reflective of what was found with active surveillance [41]. Large outbreaks of a particular serotype could affect the analysis of concentration by geography or season. However, by limiting the analysis to only the common serotypes and aggregating data over 16 years, the effect of reporting differences and single outbreaks was minimized for the discussion of general trends. As outbreak-associated cases are not identified consistently in the national surveillance system, and as periodic outbreaks may indeed be a part of the characteristics for some serotypes, it was neither practical nor desirable to exclude cases known to be outbreak-associated in this paper. Future investigations into specific serotypes may be well served by examining the serotype-specific characteristics with and without outbreak-related cases included.

This study illustrates the important differences among and complexity of *Salmonella* serotypes. These differences, and the continual increase in incidence in some serotypes while other serotypes have a simultaneous decreasing incidence, call for a better understanding of individual serotypes. Application of the CV of incidence rates as an organizing principle for *Salmonella* serotypes provides an easy and efficient way to identify serotypes concentrated by different epidemiological variables. Variability of the incidence of *Salmonella* infections by serotype with respect to epidemiological variables could inform our understanding of the overall percentage of *Salmonella* infection that is foodborne. Without such understanding one cannot judge the effect of preventive measures. For example, to estimate the percentage of infections that are foodborne, one exploratory hypothesis that could be explored is that generally uniform incidence points primarily to foodborne causes while highly localized incidence points to other causes. Deeper investigation into specific serotypes will help to support or refute such hypotheses and better our understanding of *Salmonella* in order to develop more targeted prevention efforts.

## Supporting Information

S1 Table Coefficient of Variance for *Salmonella* Serotypes Reported at Least 1,600 Times During 1996–2011 to CDC, by State, Month of Year, Age Group, and Sex, with Illustrative Figures (listed from most to least reported). (DOCX)
