# Supplementary material for: Salmonella enterica Infections in the United States and Assessment of Coefficients of Variation: A Novel Approach to Identify Epidemiologic Characteristics of Individual Serotypes, 1996–2011
Source: PLoS One. 2015 Dec 23;10(12):e0145416. doi: 10.1371/journal.pone.0145416 (PMC4689500; doi:10.1371/journal.pone.0145416)
Supplement: S1 Table — (DOCX) [file pone.0145416.s001.docx]

**Supplemental Table 1. Coefficient of Variance for *Salmonella* Serotypes Reported at Least 1,600 Times During 1996-2011 to CDC, by State, Month of Year, Age Group, and Sex, with Illustrative Figures (listed from most to least reported)**

| SerotypeNo. of Isolates,^d^Rate,^e^% Change^f^ | CV,^a^ Distribution by State^b^(# Isolates/100,000 Persons by State)^g^ | CV,^a^ Distribution by Month(% Isolates by Month) | CV,^a^ Distribution by Age^c^(# Isolates/100,000 Persons by Age group) | CV,^a^ Distribution by Sex(% Isolates by Sex) |
| --- | --- | --- | --- | --- |
| Typhimurium^+ h^ | 29.8 | 31.1 | 95 | 4.8 |
| 126,528  2.73  -16% | 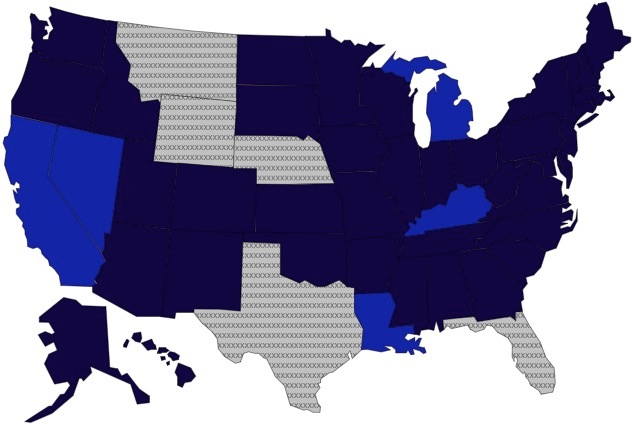 | 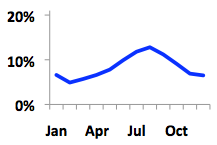 | 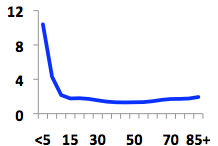 | 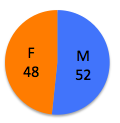 |
| Enteritidis | 44.7 | 29.1 | 24.4 | 5.6 |
| 106,514  2.29  -1.1% | 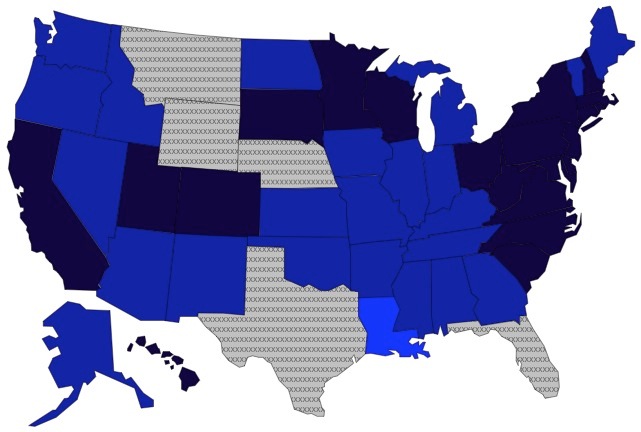 | 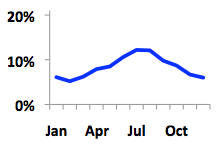 | 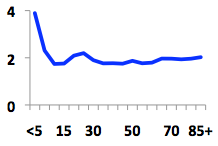 | 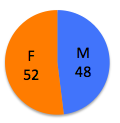 |
| Newport | 78.3 | 68.1 | 80.8 | 9.6 |
| 54,487  1.17  +26.8% | 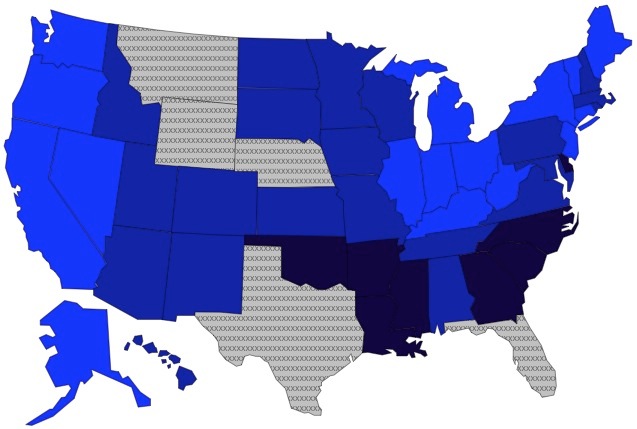 | 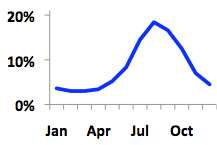 | 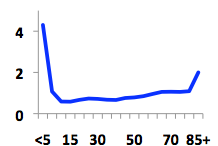 | 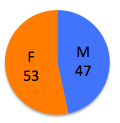 |
| Heidelberg | 33.1 | 31.7 | 78.8 | 0.3 |
| 26,980  0.88  -30% | 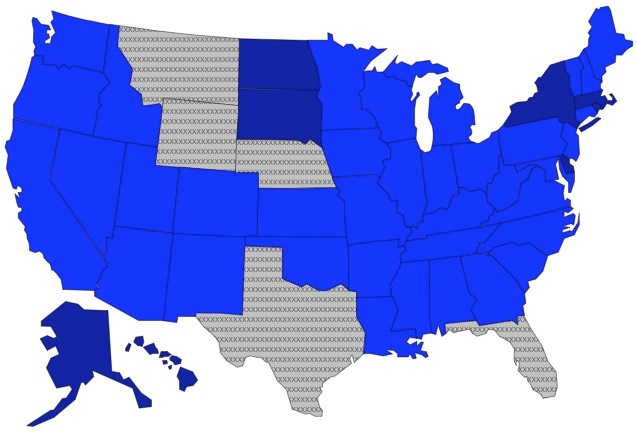 | 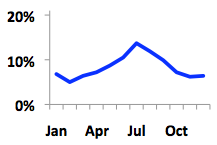 | 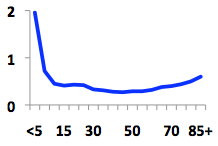 | 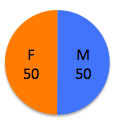 |
| Javiana | 135.1 | 82.6 | 115.9 | 1.5 |
| 24,936  0.54  +63.1% | 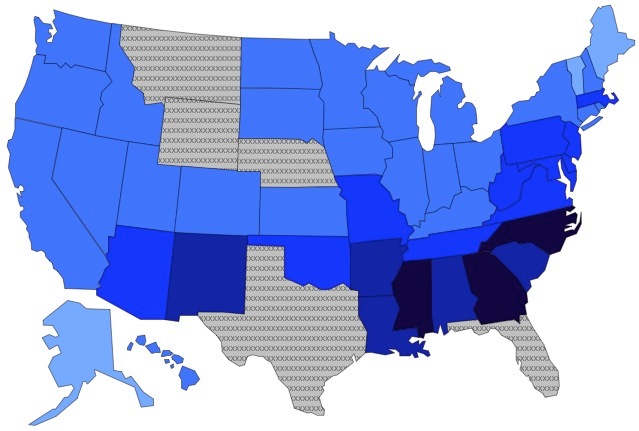 | 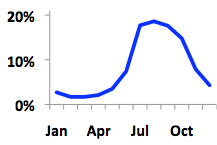 | 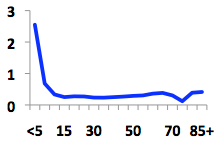 | 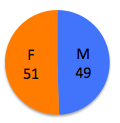 |

| SerotypeNo. of Isolates,^d^Rate,^e^% Change^f^ | CV,^a^ Distribution by State^b^(# Isolates/100,000 Persons by State)^g^ | CV,^a^ Distribution by Month(% Isolates by Month) | CV,^a^ Distribution by Age^c^(# Isolates/100,000 Persons by Age group) | CV,^a^ Distribution by Sex(% Isolates by Sex) |
| --- | --- | --- | --- | --- |
| Montevideo | 57.8 | 38 | 80.5 | 10.1 |
| 15,080  0.43  +13.7% | 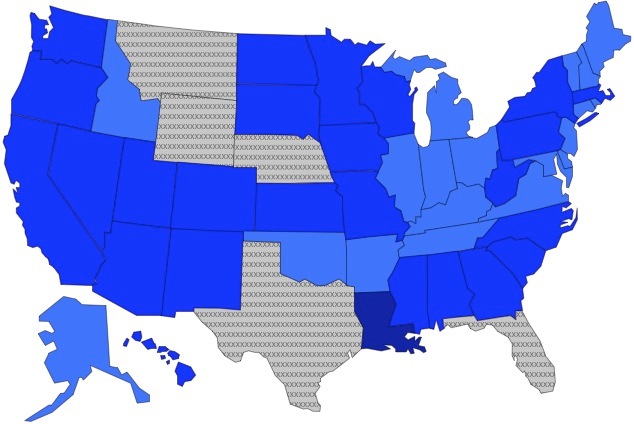 | 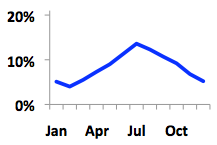 | 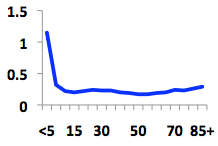 | 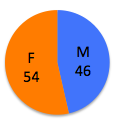 |
| Muenchen | 95.8 | 61.3 | 93.6 | 12.3 |
| 12,465  0.32  +7.4% | 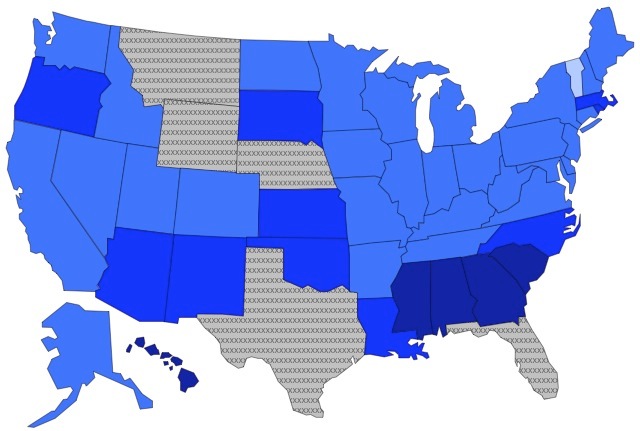 | 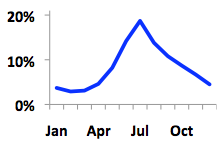 | 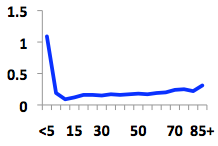 | 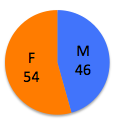 |
| Saintpaul | 53.5 | 41.6 | 67.7 | 4.6 |
| 11,126  0.24  +42.8% | 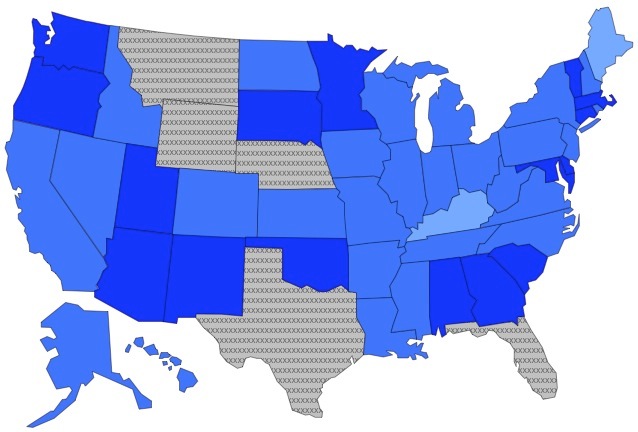 | 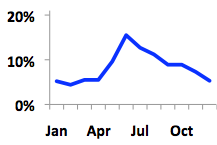 | 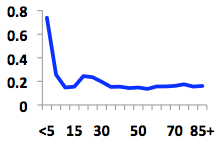 | 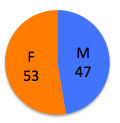 |
| Oranienburg | 76.3 | 38 | 68.5 | 10.1 |
| 10,413  0.22  -0.3% | 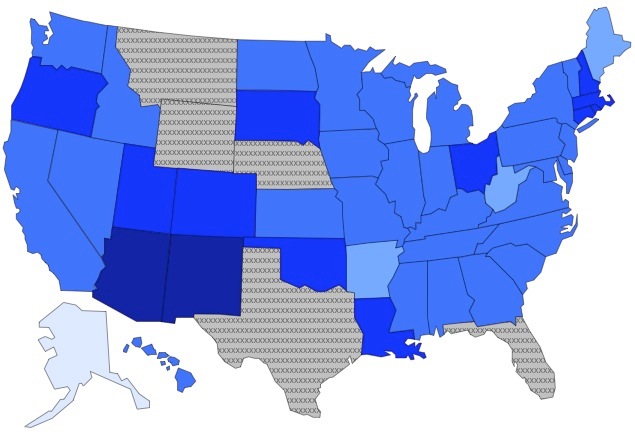 | 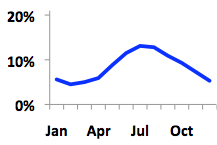 | 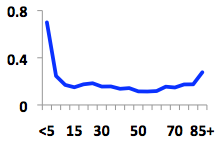 | 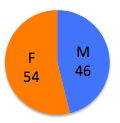 |
| Infantis | 39.8 | 33.1 | 63.3 | 11.3 |
| 9,566  0.21  +5.0% | 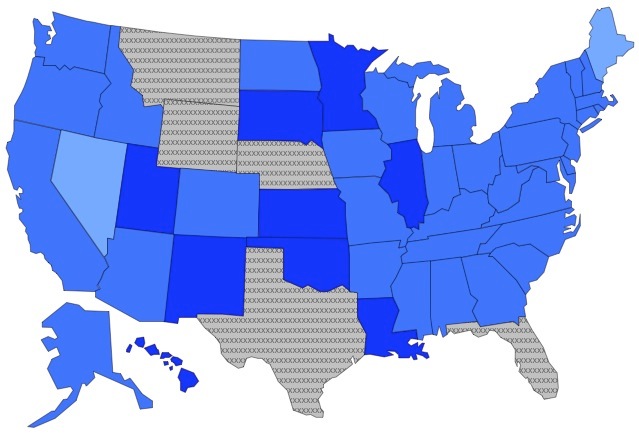 | 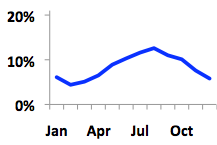 | 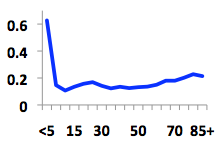 | 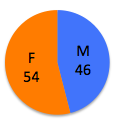 |
| Braenderup | 40.8 | 38 | 41.3 | 16.1 |
| 9,270  0.20  +21.2% | 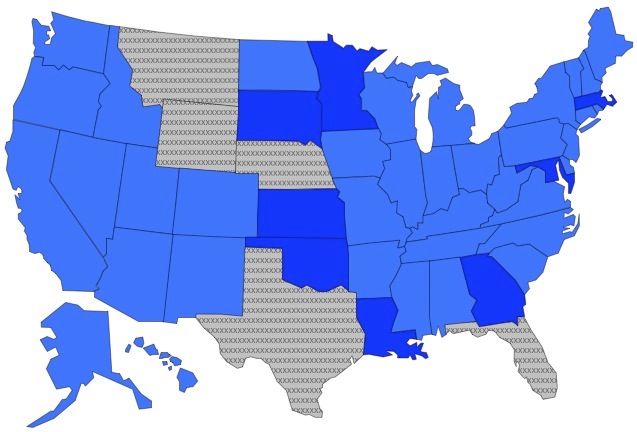 | 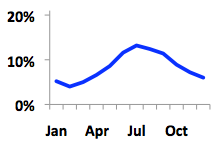 | 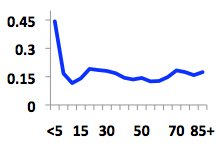 | 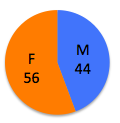 |

| SerotypeNo. of Isolates,^d^Rate,^e^% Change^f^ | CV,^a^ Distribution by State^b^(# Isolates/100,000 Persons by State)^g^ | CV,^a^ Distribution by Month(% Isolates by Month) | CV,^a^ Distribution by Age^c^(# Isolates/100,000 Persons by Age group) | CV,^a^ Distribution by Sex(% Isolates by Sex) |
| --- | --- | --- | --- | --- |
| Paratyphi B. Var L(+) Tartrate+ ^i^ | 65.7 | 32.1 | 92.9 | 6.2 |
| 8,585  0.17  0% | 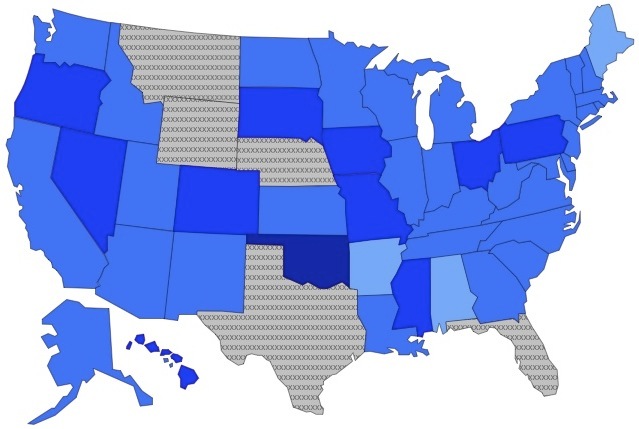 | 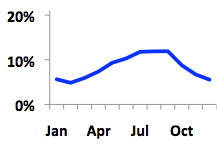 | 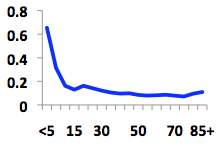 | 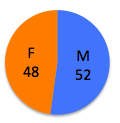 |
| Agona | 46 | 32.3 | 72 | 4.5 |
| 8,348  0.18  -22.6% | 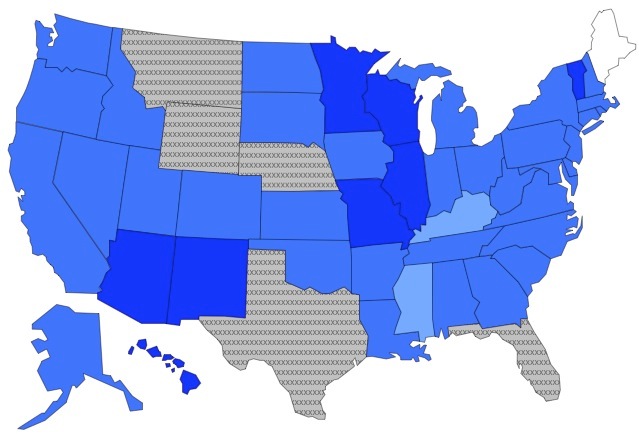 | 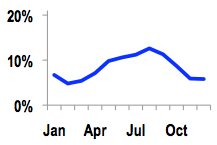 | 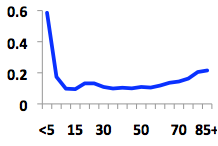 | 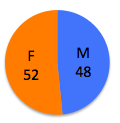 |
| Thompson | 44.6 | 42.2 | 49.6 | 11.9 |
| 8,225  0.18  -25.7% | 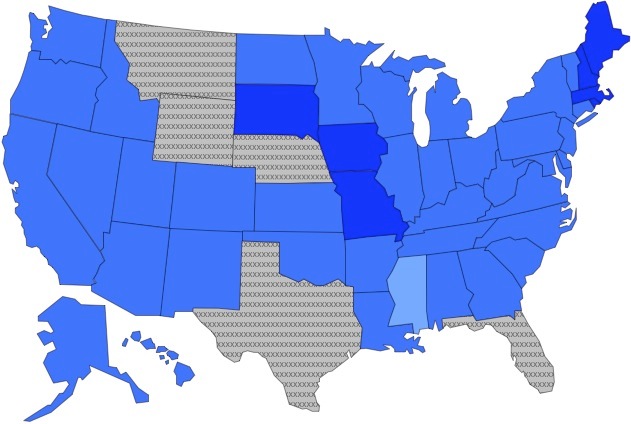 | 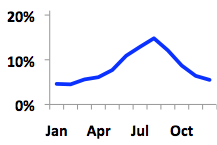 | 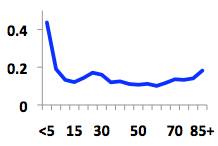 | 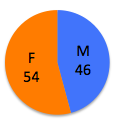 |
| Mississippi | 255.8 | 69.3 | 160.1 | 7.7 |
| 6,438  0.14  +62.5% | 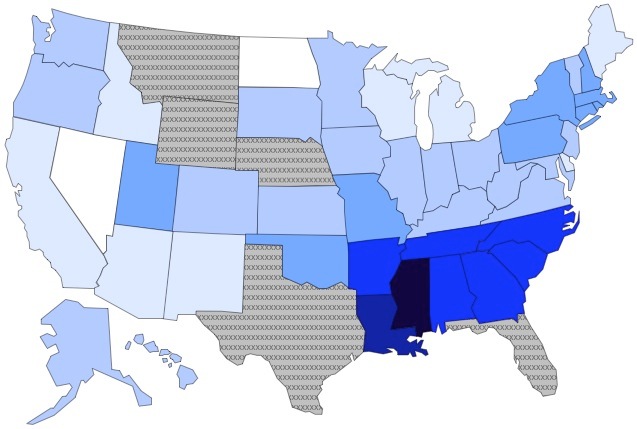 | 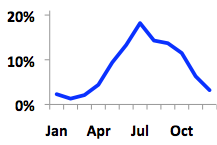 | 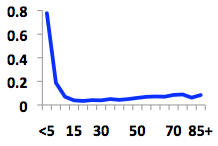 | 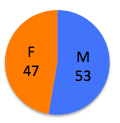 |
| Typhi | 93.1 | 28.7 | 67.8 | 6 |
| 6,211  0.13  +2.9% | 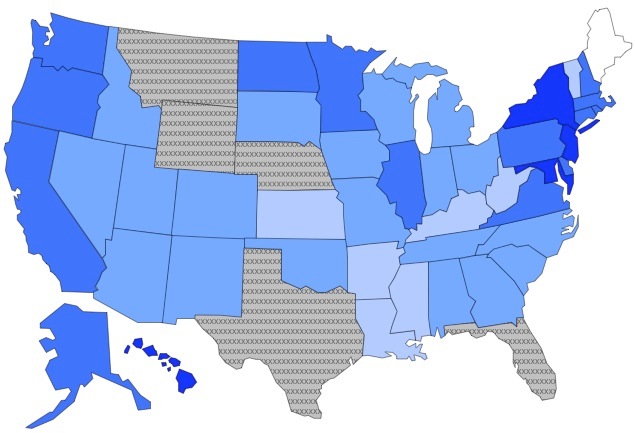 | 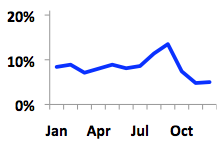 | 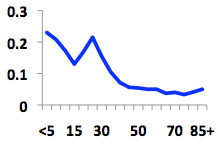 | 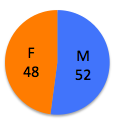 |
| Hadar | 58.8 | 27.4 | 52 | 8.6 |
| 5,683  0.12  -48.8% | 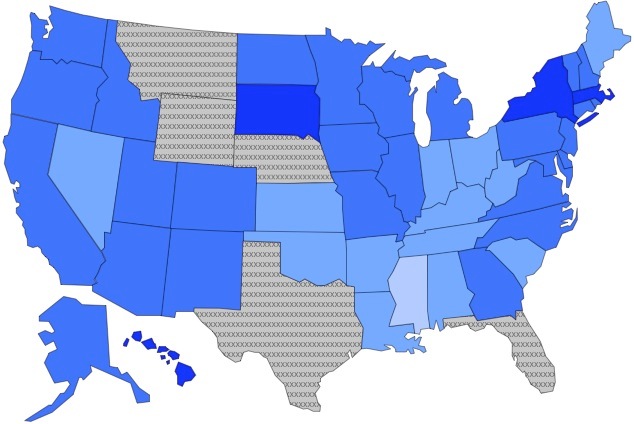 | 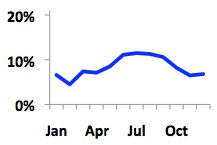 | 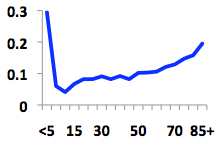 | 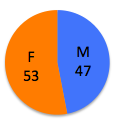 |
| SerotypeNo. of Isolates,^d^Rate,^e^% Change^f^ | CV,^a^ Distribution by State^b^(# Isolates/100,000 Persons by State)^g^ | CV,^a^ Distribution by Month(% Isolates by Month) | CV,^a^ Distribution by Age^c^(# Isolates/100,000 Persons by Age group) | CV,^a^ Distribution by Sex(% Isolates by Sex) |
| Poona | 107.2 | 28.7 | 151 | 3.5 |
| 4,645  0.10  -19.5% | 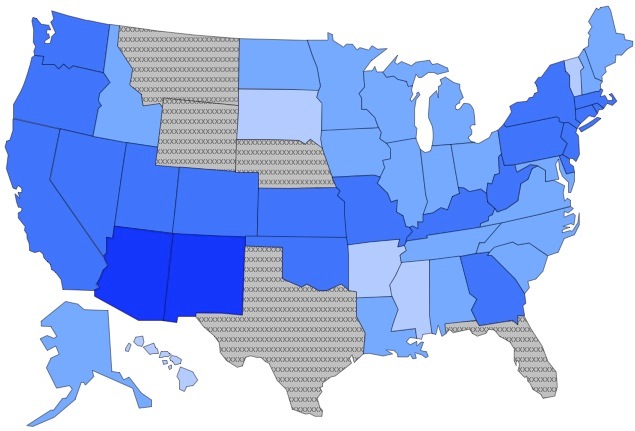 | 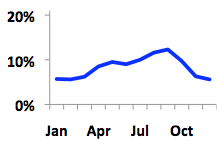 | 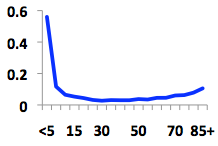 | 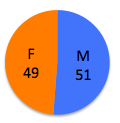 |
| Berta | 60.1 | 56.7 | 40.3 | 10.3 |
| 3,635  0.08  +15.9% | 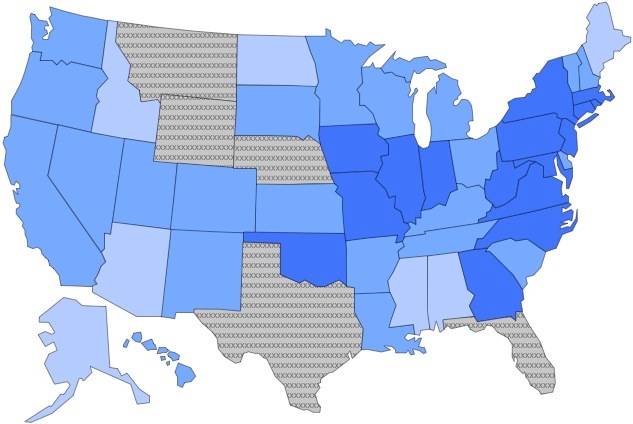 | 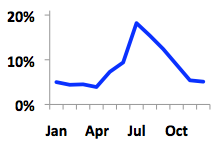 | 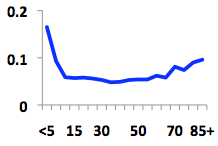 | 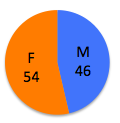 |
| Bareilly | 127 | 63.3 | 100.1 | 1.3 |
| 3,576  0.08  +49.5% | 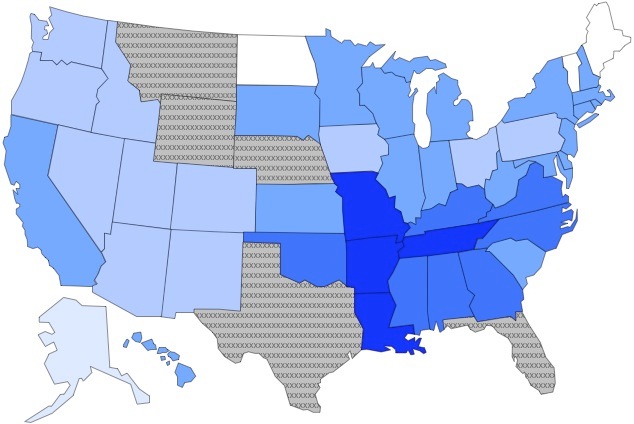 | 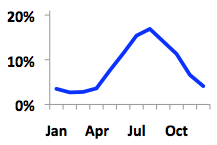 | 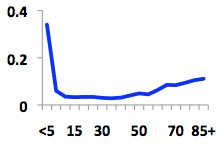 | 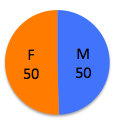 |
| Stanley | 54.3 | 27 | 129 | 9.1 |
| 3,357  0.07  +7.8% | 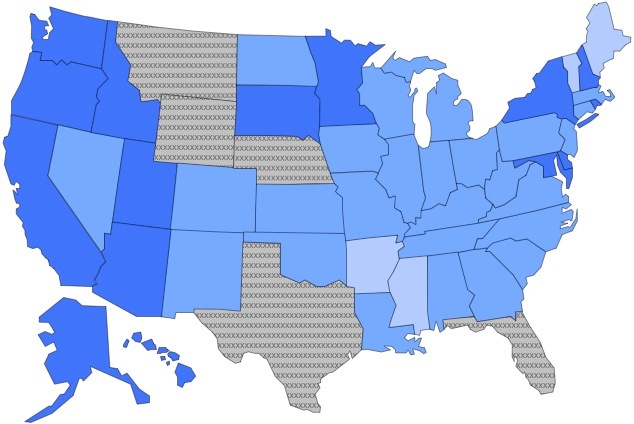 | 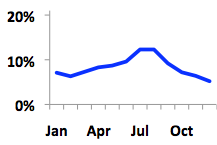 | 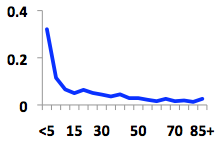 | 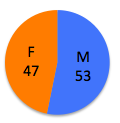 |
| Anatum | 56.6 | 24 | 49 | 27.8 |
| 3,336  0.07  +7.6% | 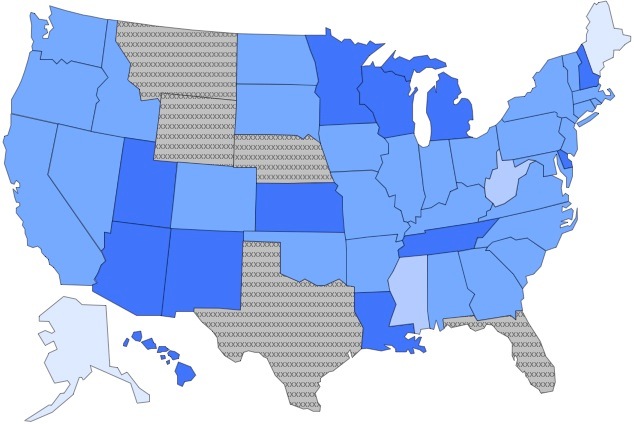 | 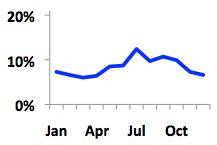 | 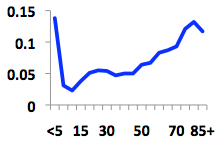 | 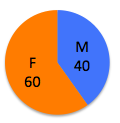 |
| Schwarzengrund | 92.7 | 42.3 | 149.7 | 0.6 |
| 3,185  0.07  +78.1% | 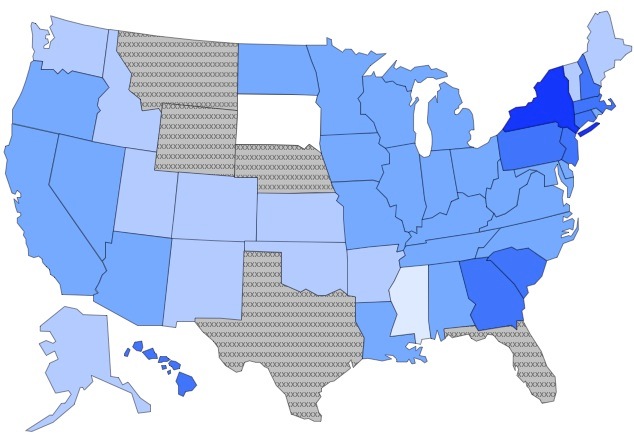 | 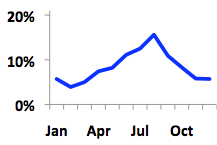 | 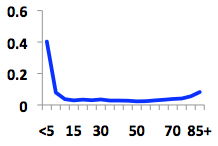 | 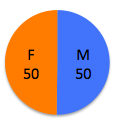 |
| SerotypeNo. of Isolates,^d^Rate,^e^% Change^f^ | CV,^a^ Distribution by State^b^(# Isolates/100,000 Persons by State)^g^ | CV,^a^ Distribution by Month(% Isolates by Month) | CV,^a^ Distribution by Age^c^(# Isolates/100,000 Persons by Age group) | CV,^a^ Distribution by Sex(% Isolates by Sex) |
| Mbandaka | 58.5 | 20.8 | 70.8 | 23.6 |
| 3,159  0.07  +7.1% | 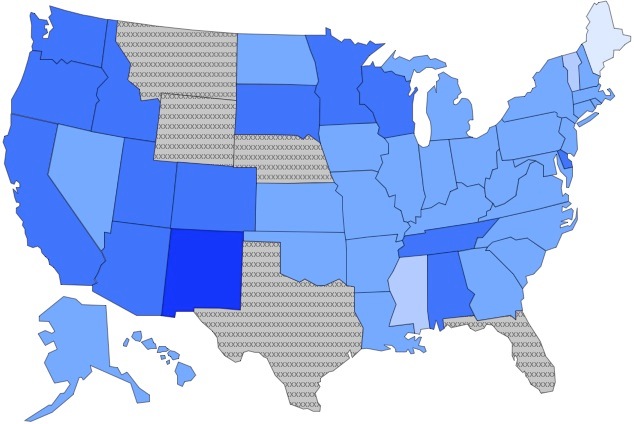 | 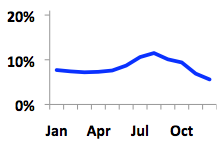 | 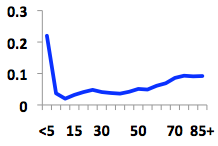 | 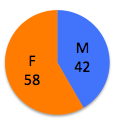 |
| Hartford | 73.2 | 56.7 | 43.2 | 14.1 |
| 2,921  0.06  +30.7% | 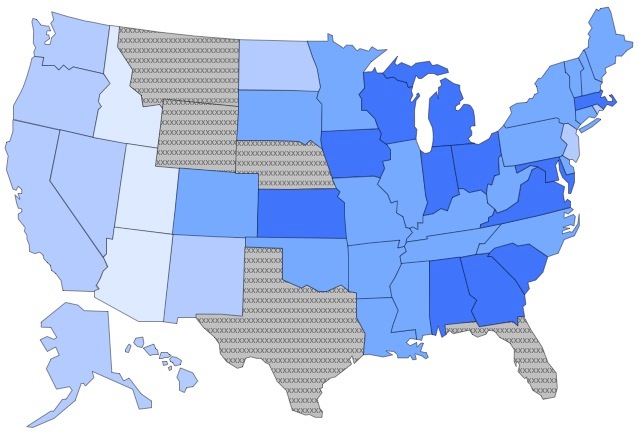 | 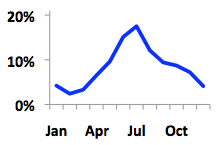 | 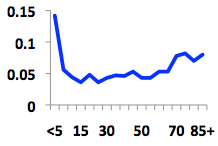 | 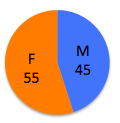 |
| Litchfield | 78.3 | 39.1 | 70.6 | 14.3 |
| 2,839  0.06  +51.5% |  |  |  |  |
| Panama | 101.2 | 34.5 | 67.6 | 7.4 |
| 2,584  0.06  +5.9% |  |  |  |  |
| Derby | 105.9 | 24 | 88 | 5.7 |
| 2,312  0.05  -21.2% |  |  |  |  |
| Senftenberg | 76.1 | 18.3 | 49.8 | 34.7 |
| 2,247  0.05  -9.6% |  |  |  |  |
| SerotypeNo. of Isolates,^d^Rate,^e^% Change^f^ | CV,^a^ Distribution by State^b^(# Isolates/100,000 Persons by State)^g^ | CV,^a^ Distribution by Month(% Isolates by Month) | CV,^a^ Distribution by Age^c^(# Isolates/100,000 Persons by Age group) | CV,^a^ Distribution by Sex(% Isolates by Sex) |
| Sandiego | 64.1 | 29.5 | 91.8 | 0.5 |
| 2,050  0.04  +43.3% |  |  |  |  |
| Paratyphi A | 80.6 | 34.6 | 62.5 | 13.7 |
| 2,043  0.04  +70.6% |  |  |  |  |
| Brandenburg | 59 | 26.5 | 85.3 | 1.6 |
| 1,722  0.04  -40.6% |  |  |  |  |
| Tennessee | 48.7 | 32.6 | 73.2 | 53.5 |
| 1,721  0.04  +253% |  |  |  |  |
| Norwich | 161.2 | 87 | 111.3 | 5.6 |
| 1,683  0.04  +49.7% |  |  |  |  |
| Rubislaw | 197.3 | 56.3 | 264.6 | 7.3 |
| 1,629  0.04  +32.2% |  |  |  |  |
| SerotypeNo. of Isolates,^d^Rate,^e^% Change^f^ | CV,^a^ Distribution by State^b^(# Isolates/100,000 Persons by State)^g^ | CV,^a^ Distribution by Month(% Isolates by Month) | CV,^a^ Distribution by Age^c^(# Isolates/100,000 Persons by Age group) | CV,^a^ Distribution by Sex(% Isolates by Sex) |
| Give | 195.1 | 45.1 | 96.5 | 9.6 |
| 1,611  0.03  +9.7% |  |  |  |  |
| All Others | 59.4 | 28.1 | 89.2 | 5.9 |
| 50,455  1.09  +17.5% |  |  |  |  |

^a^ CV = Coefficient of variation.

^b^ Excludes 6 states reporting >10% of isolates with no serotype data or only partial serotype data for 8 or more of the 16 surveillance years (shown in gray on maps: Montana, Texas, Florida, Nebraska, Wyoming, Washington DC).

^c^  Age groups based on US census age groups of 18 groupings (6 of the 18 age groups shown on x-axis due to space).

^d^ Total isolates reported during 1996-2011 in the United States.

^e^ Rate of isolates (# per 100,000 persons) in the United States during 1996-2011.

^f^ Percentage change in isolation rate (per 100,000 persons) during 2004-2011 compared with 1996-2003.

^g^ Legend (no. of isolates per 100,000 population):

^h^ Includes serotype I, 4,[5],12:i:- and Typhimurium isolates reported as Typhimurium var. 5-.

^i^ Includes isolates reported as Paratyphi B.
